# Correlation between Peripheric Blood Markers and Surgical Invasiveness during Humeral Shaft Fracture Osteosynthesis in Young and Middle-Aged Patients

**DOI:** 10.3390/diagnostics14111112

**Published:** 2024-05-27

**Authors:** Flaviu Moldovan

**Affiliations:** Orthopedics—Traumatology Department, Faculty of Medicine, “George Emil Palade” University of Medicine, Pharmacy, Science, and Technology of Targu Mures, 540142 Targu Mures, Romania; flaviu.moldovan@umfst.ro; Tel.: +40-754-671-886

**Keywords:** humeral fractures, internal fracture fixation, biomarkers, inflammation

## Abstract

The treatment for humeral shaft fractures (HSFs) is still controversial, consisting of a wide variety of orthopedic osteosynthesis materials that imply different grades of invasiveness. The aim of this study is to investigate the correlation between inflammatory blood-derived markers and the magnitude of the surgical procedure in young and middle-aged patients who sustained these fractures. Observational, retrospective research was conducted between January 2018 and December 2023. It followed patients diagnosed with recent HFSs (AO/OTA 12−A and B) and followed operative treatment. They were split in two groups, depending on the surgical protocol: group A, operated by closed reduction and internal fixation (CRIF) with intramedullary nails (IMNs), and group B, operated by open reduction and internal fixation (ORIF) with dynamic compression plates (DCPs). Statistically significant differences (*p* < 0.05) between the two groups could be observed in injury on the basis of surgery durations, surgical times, pre- and postoperative neutrophil-per-lymphocyte ratio (NLR), postoperative platelet-per-lymphocyte ratio (PLR), monocyte-per-lymphocyte ratio (MLR), systemic inflammatory index (SII), systemic inflammatory response index (SIRI) and aggregate inflammatory systemic index (AISI). The multivariate regression model proposed revealed that NLR > 7.99 (*p* = 0.007), AISI > 1668.58 (*p* = 0.008), and the surgical times (*p* < 0.0001) are strongly correlated to the magnitude of the surgical protocol followed. Using receiver operating characteristic (ROC) curve analysis, a balanced reliability was determined for both postoperative NLR > 7.99 (sensitivity 75.0% and specificity 75.6) and AISI > 1668.58 (sensitivity 70.6% and specificity 82.2%). Postoperative NLR and AISI as inflammatory markers are highly associated with the magnitude of surgical trauma sustained during humeral shaft fracture osteosynthesis in a younger population.

## 1. Introduction

Humeral shaft fractures (HSFs) are one of the most frequent upper-limb injuries, representing around 5% of all types of fractures [1]. Although these fractures are commonly seen in young patients under 30 due to the high-energy mechanism of the injury, a bimodal age distribution was reported, with the overall incidence being up to 15/100.000 [2]. Simple fracture patterns (Arbeitsgemeinschaft für Osteosynthesefragen/Orthopedic Trauma Association—AO/OTA 12−A and B) account for over 60% of cases, according to recent studies [3,4].

Surgical treatment options remain controversial with these injuries, as conservative management offers good functional outcomes, and union rates above 90% are expected [5]. However, many studies tried to compare the most common osteosynthesis modalities available: the dynamic compression plate (DCP) and the antegrade intramedullary nail (IMN) [6,7,8]. A general agreement is still lacking, because IMNs are not yet considered a “gold standard” technique for the treatment of HSFs like in the case of closed femoral or tibial shaft fractures, as they are associated with higher rates of reoperation, degrees of malrotation and shoulder impingement with decreased range of movement [7]. The special biomechanical characteristics of the arm make DCPs an excellent alternative, although they require an extensive procedure with soft tissue stripping from the bone, radial nerve complications due to interposition into the area of dissection and a more unstable fixation in osteopenic patients [9].

Tissue damage due to surgical trauma in the acute postoperative period produces a local and systemic inflammatory state. This represents a physiological response in the wound-healing process, also contributing to anti-pathogen defense mechanisms [10]. According to Amodeo et al. [11], immediately after major surgeries, an impaired immune function can be seen with reduction in lymphoproliferation, TNF-α, IL-2 and IFN-γ. Just a few hours after any surgical or accidental trauma, damage response antigens and alarmins rapidly recruit and activate neutrophils and monocytes [12]. This initial leukocyte “rolling” process is accompanied with platelet (PLT) adhesions, which leads to white blood cell (WBC) extravasations to the inflammation or infection sites [13].

Recently, novel inflammatory markers derived from peripheral blood counts have been proposed due to the ease and frequency of this investigation. The neutrophil-per-lymphocyte ratio (NLR), platelet-per-lymphocyte ratio (PLR) and monocyte-per-lymphocyte ratio (MLR) have been linked to different clinical outcomes of various diseases such as pneumonia, acute myocardial infarction and cancer [14,15,16]. They also proved their efficiency through their low values in predicting a better survival after major interventions, including in lung cancer patients [17]. The need to have a more general overview of the inflammatory response led to the development of more complex markers. For example, Song et al. [18] suggested that higher levels of the systemic inflammatory index (SII), the aggregate inflammatory systemic index (AISI) and the systemic inflammatory response index (SIRI) correlate independently with the clinical severity in type 2 diabetic patients with peripheral arterial disease. Other studies [19,20] demonstrated their utility in pulmonary embolism for risk stratification.

Up to present, there have been no studies on the possible use of NLR, PLR, MLR, SII, AISI and SIRI in quantifying the invasiveness of HSF surgical protocols. Therefore, this retrospective study aims to analyze the relation between these dynamic inflammatory markers and the magnitude of the surgical procedure in young and middle-aged patients with HFSs.

## 2. Materials and Methods

### 2.1. Study Design and Patient Selection

**Figure 1:**
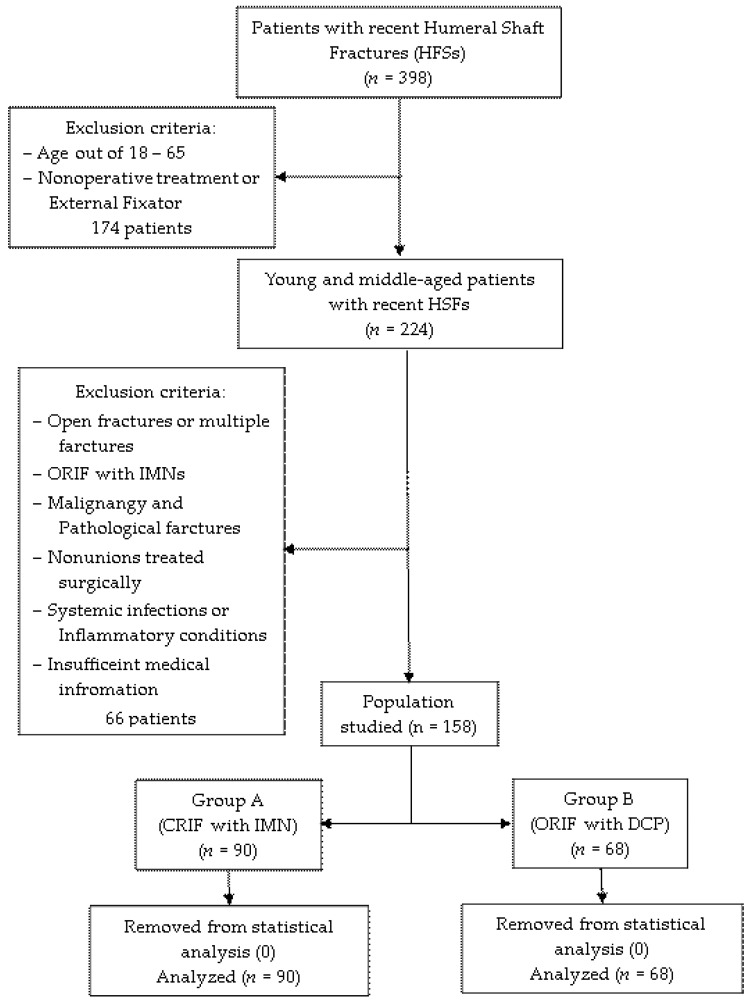
Selection process of the studied population.

All data were obtained and analyzed in a retrospective method from the electronic database of Mures County Emergency Hospital in Targu Mures, Romania at the Orthopedics-Traumatology Department between January 2018 and December 2023. The study was approved by the Hospital’s Ethics Committee (protocol code Ad.22522/17.09.2021). Young and middle-aged patients with recent humeral shaft fractures who underwent surgical treatment with intramedullary nailing (IMN) or dynamic compression plate (DCP) were included in the study. Exclusion criteria were as follows: (1) age < 18 and >65 years; (2) nonoperative treatment or external fixators; (3) open fractures or multiple fractures; (4) fractures treated with IMNs that could not be reduced by closed means, and conversion to open reduction was mandatory during surgery; (5) malignancy and pathological fractures; (6) fracture nonunion that eventually were operated; (7) associated systemic infections or inflammatory conditions; (8) insufficient medical information. Initially, the patients were divided according to the surgical protocol followed: group A, operated by closed reduction and internal fixation (CRIF) with IMNs (*n* = 90), and group B, operated by open reduction and internal fixation (ORIF) with DCPs (*n* = 68). All 158 cases were further included in the statistical analysis, as no measurement errors, data entries, processing failures or poor samplings were identified. Figure 1 provides a detailed flowchart of the selection process.

### 2.2. Data Extraction

The following variables were extracted for statistical processing: (1) age, sex and residence; (2) health risk behaviors such as smoking, alcohol consumption and overweight (BMI ≥ 25); (3) associated medical conditions (hypertension, IHD—ischemic heart disease; asthma; CB—chronic bronchitis; type 2 diabetes); (4) fracture characteristics, including AO classification, mechanism of injury (MoI) and side of the fracture; (5) surgical parameters such as the American Society of Anesthesiologists (ASA) score, type of anesthesia, time from injury till surgery, length of hospitalization (LOH) and surgery duration; (6) complete blood count (CBC) data in the pre- and postoperative period: neutrophil (N) counts, lymphocyte (L) counts, monocyte (M) counts, platelet (PLT) counts, aspartate–transaminase/alanine–transaminase (AST/ALT) ratios, white blood counts (WBC), red blood counts (RBC) and hemoglobin (HB) levels.

### 2.3. Dynamic Markers of Inflammation

With the scope of evaluating and quantifying the pre- and postoperative dynamics of the inflammation process in relation to the magnitude of the two operative protocols proposed, the following markers were computed from CBCs: (1) neutrophil-per-lymphocyte ratio (NLR), with the formula NLR = N/L; (2) platelet-per-lymphocyte ratio (PLR), with the formula PLR = PLT/L; (3) monocyte-per-lymphocyte ratio (MLR), with the formula MLR = M/L; (4) systemic inflammatory index (SII), with the formula SII = [N × PLT]/L; (5) systemic inflammatory response index (SIRI), with the formula SIRI = [M × PLT]/L; (6) the aggregate inflammatory systemic index (AISI), with the formula AISI = [N × M × PLT/L].

### 2.4. Surgical Protocols

The following two protocols were employed by experienced orthopedic physicians from the clinic, depending on their preference and particularities of the cases at the time of surgery. Patient positioning was performed in a supine or the “beach chair” manner with the head fixed to the contralateral side of the injury. For the participants in group A (CRIF with IMNs), an antegrade nailing technique using a bent proximal nailing system with targeting devices was performed in all cases. The incision length was approximately 2−3 cm from the anterolateral margin of the acromion, aiming the insertion of the deltoid obliquely. For the participants in group B (ORIF with DCPs), osteosynthesis was achieved with traditional 4.5 mm narrow dynamic compression plates with staggered screws. Depending on the fracture configuration and its location, either anterolateral (proximal and middle shaft fractures) or posterior (distal shaft fractures) approaches were used. The incisions surpassed 12 cm in length in all cases.

### 2.5. Statistical Steps

Statistical analysis was performed using IBM SPSS, version 29.0.2 (SPSS, Inc., Chicago, IL, USA) for Widows. The Shapiro–Wilk test was first carried out on all continuous variables to assess their normality check, followed by analysis with the Student’s t test or Mann–Whitney U test. Significant intergroup variations were determined with the Chi-square test or Fisher’s exact test for the categorical variables. The cut-off values, area under the curve (AUC), sensitivity and specificity of the relevant ratios and scale data were identified by receiver operating characteristic (ROC) curve analysis, based on Youden’s index (Youden index = sensitivity + specificity − 1, with a range between 0–1). Statistically significant variables (*p* value < 0.05) that presented the potential of being independent elements for quantifying the magnitude of the surgical protocol were further introduced in a fitting logistic multivariate regression model (*p* value > 0.05 of the Hosmer–Lemeshow test). The associations were investigated in terms of strength by odds ratios (OR) with a confidence interval (CI) set at 95%.

## 3. Results

In the 5-year study period, a sum of 158 young and middle-aged patients (56.3% females with the mean age of 45) with recent humeral shaft fractures (62.7% due to high mechanism of the injury) that underwent operative treatment were included. They were divided into two groups according to the type of surgical protocol followed: group A, which included 90 patients (56.96%) who preceded with CRIF (using IMNs as osteosynthesis material), and group B, which included 68 patients (43.05%) who preceded with ORIF (using DCPs as osteosynthesis material).

To better quantify the inflammatory response, a ROC curve analysis was used to determine the optimum cut-off (Table 1) values of the pre- and postoperative proposed markers (NLR, MLR, PLR, SII, SIRI and AISI). Continuous surgical parameters (time from injury to surgery, LOH and surgery duration) contributing to the general trauma sustained were assessed in the same manner.

**Figure 2:**
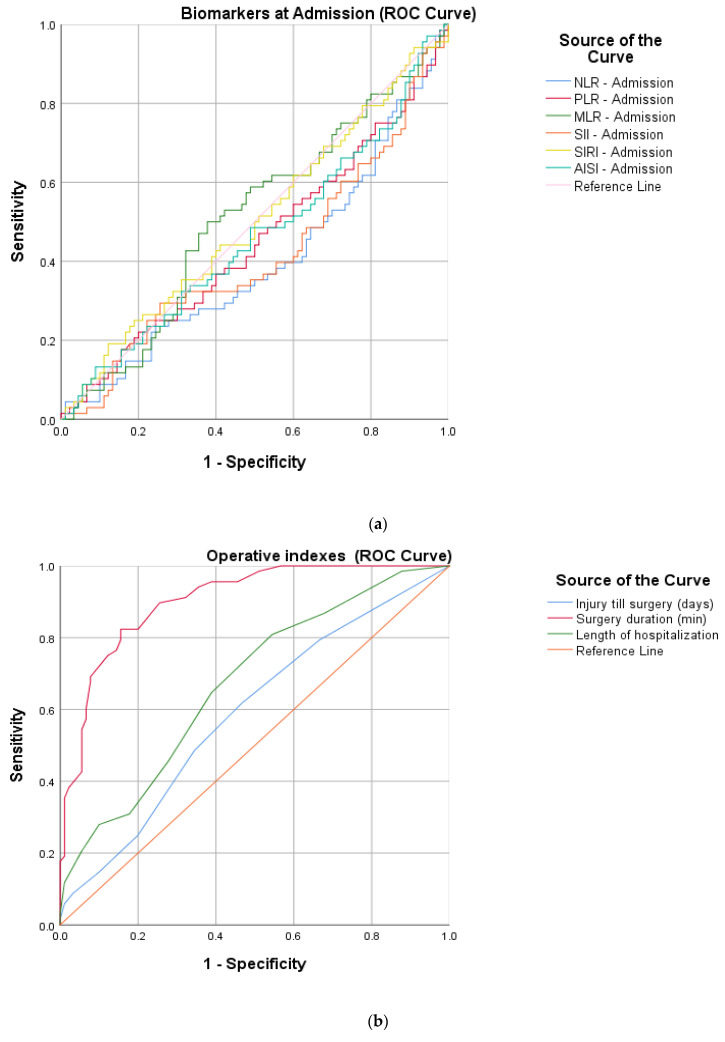

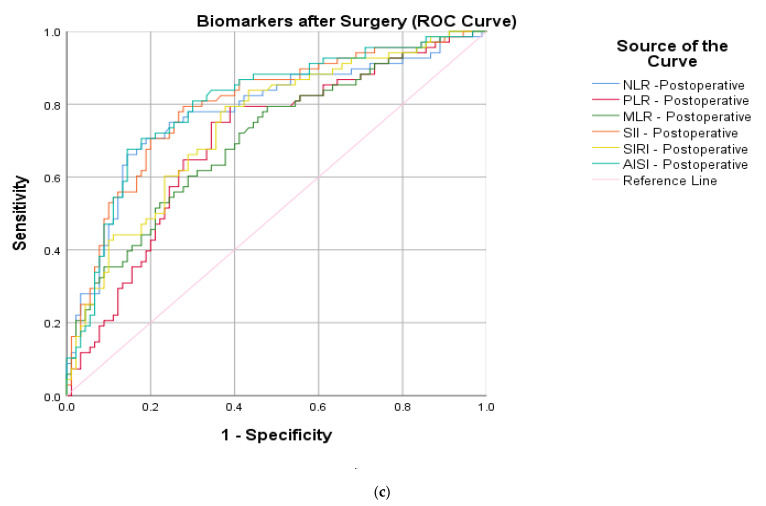
ROC curve graphs for HSF protocols indicating (**a**) peripheric blood-derived markers at admission, (**b**) surgical parameters (injury till surgery, surgery duration and length of hospitalization) and (**c**) postoperative peripheric blood-derived markers.

After calculating the sensitivity, specificity and AUC (area under the curve), an increased precision power (Figure 2) was identified for the postoperative markers and two of the surgical parameters: NLR (cut-off 7.20, sensitivity 75%, specificity 76.6%, AUC 0.780), PLR (cut-off 174.22, sensitivity 79.4%, specificity 61.1%, AUC 0.700), MLR (cut-off 0.82, sensitivity 61.8%, specificity 68.9%, AUC 0.707), SII (cut-off 1564.74, sensitivity 79.4%, specificity 72.2%, AUC 0.797), SIRI (cut-off 156.95, sensitivity 77.9%, specificity 63.3%, AUC 0.744), AISI (cut-off 1668.58, sensitivity 70.6%, specificity 82.2%, AUC 0.802), surgery duration (cut-off 61, sensitivity 82.4%, specificity 84.4%, AUC 0.904) and LOH (cut-off 6, sensitivity 80.9%, specificity 45.6%, AUC 0.673).

The following postoperative laboratory data presented statistically significant differences in the univariate analysis (Table 2): neutrophil count (*p* < 0.0001), lymphocyte count (*p* = 0.007), monocyte count (*p* = 0.010), PLT count (*p* = 0.002) and WBC (*p* < 0.0001). These results can explain the high statistical value (*p* < 0.0001) of each marker after the surgery. A slight difference (*p* = 0.045) could also be identified in preoperative NLR. Other significant variables from the baseline characters and surgical parameters included LOH (*p* = 0.004) and surgery duration (*p* < 0.0001).

**Figure 3:**
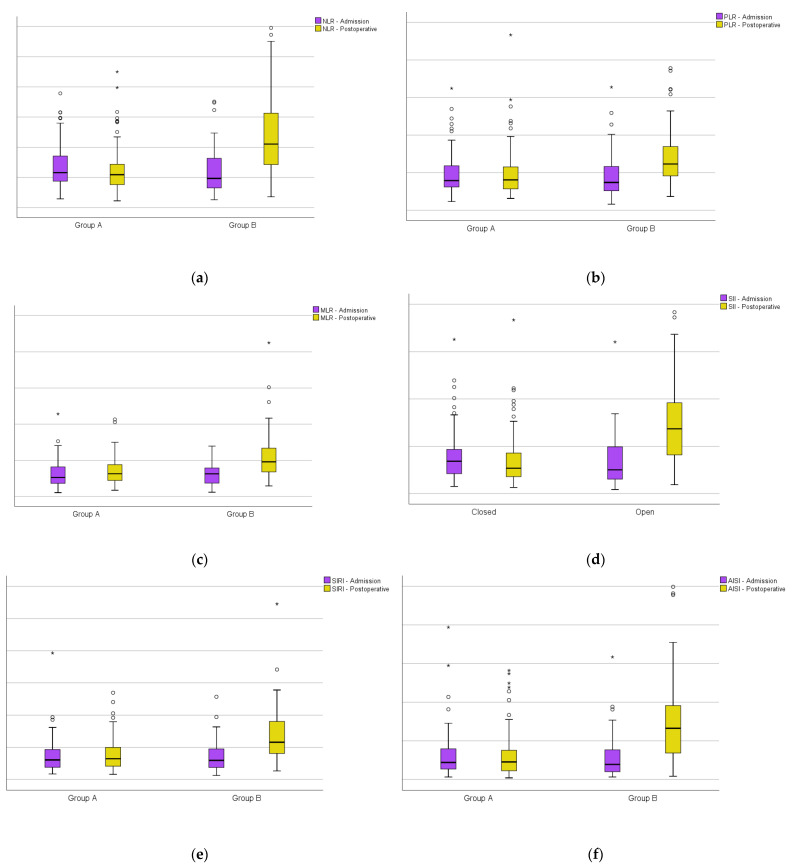
Boxplots of group A versus group B at admission and on the first day after surgery: (**a**) the neutrophil-per-lymphocyte ratio; (**b**) platelet-per-lymphocyte ratio; (**c**) monocyte-per-lymphocyte ratio; (**d**) systemic inflammatory index; (**e**) systemic inflammatory response index; (**f**) aggregate inflammatory systemic index. Circles represent the mild outliers and asterisks represent the extreme outliers.

Furthermore, in the immediate postoperative period, a significant increase can be seen in the studied markers (NLR, PLR, MLR SII, SIRI and AISI) compared to the admission period for the patients in Group B (Figure 3).

In the multivariate logistic regression (Table 3), apart from the relevant postoperative markers, surgical parameters that may influence the systemic immune response of the organism were included. The constructed equation confirmed the correlation with the magnitude of the surgical intervention through the following variables: NLR postoperative (OR 17.12, 95% CI 2.14–136.33, *p* = 0.007), AISI postoperative (OR 16.15, 95% CI 2.08–125.44, *p* = 0.008) and duration of surgery (OR 76.42, 95% CI 14.41–405.62, *p* < 0.0001). The proposed model showed a good fitness though the Hosmer–Lemeshow test (X^2^ = 11.076, *p* = 0.135 and Nagelkerke R^2^ = 0.734).

## 4. Discussion

Surgical treatment options for HSFs in terms of technique and osteosynthesis material have been long debated without reaching a consensus. Both DCPs and IMNs have their own sets of advantages and disadvantages. A recent systematic review conducted by Bergen et al. [21] that included 173 studies on the subject concluded that plates have higher fracture healing rates and the least complications if radial nerve palsies are excluded. On the contrary, a study that also took into consideration operative measurements such as surgical time and intraoperative blood loss concluded that both techniques can produce similar results, although IMN was a less invasive procedure [22]. IMNs are expected to have a lower infection rate, suggesting their superiority, but in terms of functional recovery and outcomes, they are associated with lower American Shoulder and Elbow Surgeon (ASES) scores and shoulder-elbow movement limitations [23].

Given these divergent results, the present study has a different approach towards the two protocols, proposed by comparing the degree of surgical trauma by measuring the dynamics of the proposed inflammatory markers. The immune status of the subjects after surgery could be reflected with an increased precision power by all ratios (*p* < 0.0001). The most accurate were the NLR > 7.20 (sensitivity 75.0% and specificity 75.6) and AISI > 1668.58 (sensitivity 70.6% and specificity 82.2%) due to their balanced reliability. Furthermore, the multivariate model confirmed their independency to quantify the surgical-related trauma (*p* < 0.007, respectively, *p* < 0.008). A slightly stronger association can be attributed to NLR when compared to AISI (OR: 17.12 versus OR: 16.15). This can be attributed to the neutrophils, which are the common component of these two ratios. From the univariate analysis, it can be clearly seen that from all the leucocytes, their postoperative values are highly statistically significant (*p* < 0.0001). This can be explained by the fact that neutrophils are the central actors of the inflammatory process in the early stages, as they are the most numerous circulating immune cells [24]. They also lay the ground for the macrophages in the scope of repairing the tissue damage and attract a “cytokine storm”, which is characterized by a massive production of pro-inflammatory agents such as interleukin (IL)-6, IL- 1β and tumor necrosis factor alfa (TNF-α) [25]. This pro-inflammatory process after major surgeries is known as systemic inflammatory response syndrome (SIRS), which is a physiological state and is beneficial for wound healing and restoring homeostasis [26]. However, if persistent stress is present, such as infection, malnutrition or re-operations, the usual anti-inflammatory agents [s-TNF-R1, IL-4, IL-10, IL-1 receptor antagonist (IL-1Ra), transforming growth factor beta (TGF-β)] are dysregulated; thus, the compensatory anti-inflammatory immune response (CARS) may produce adverse effects [27]. One study [28] even demonstrated a faster kinetic pattern than serum C-reactive protein (CRP) levels for the NLR after total hip and knee arthroplasties. Another interesting finding is that the preoperative NLR > 4.99 was also statistically significant (*p* < 0.045) between the two groups. The mean age in this study is 45.39 ± 11.90, with a slightly younger population in the CRIF group. Immuno-aging represents a phenomenon in which there is a decreased white cell activation, together with a dysregulated posttraumatic inflammatory response in the elderly [29]; thus, similar studies that were focused on this age group did not identify this change [30,31].

In recent years, there has been an expanding interest in the domain of orthopedics-traumatology for cost-effective and easy-to-obtain inflammatory blood-derived ratios [32,33]. More specifically, one study [34] demonstrated the discriminatory ability of SII, NLR and PLR to predict mortality in patients undergoing hemiarthroplasty. Another study that also included the MLR attributed a diagnostic value to these markers for tibial shaft-related infections [35]. According to Niu et al. [36], the risk of deep vein thrombosis (DVT) can also be explored in hip fracture patents by computing them. As seen in these examples, the tendency is to correlate the markers with postoperative complications, morbidity and morality rates. Few studies, however, have tried to provide an indication for a specific surgical protocol using this perspective [37]. Wang et el. [38] examined the postoperative inflammatory process and tried to provide an answer related to this manner by comparing standard locking plates and minimally invasive percutaneous plate osteosynthesis (MIPO) in the case of bicondylar tibial plateau fractures.

As a secondary endpoint for this study, group B (treated by ORIF with DCPs) had longer surgery durations (*p* < 0.0001) and LOHs (*p* = 0.004). Procedures that lasted over 61 min were independently associated (OR 76.42, 95% CI 14.41–405.62, *p* < 0.0001) with a higher degree of the surgical-related trauma. These metrics were previously studied with the scope of measuring the magnitude of different procedures [39]. In a meta-analysis with 531 articles, a comparison between the safety of minimally invasive surgery (MIS) versus open orthopedic interventions was performed using the overall complications, pain and functional scores, hospital stays, operation time and reoperation rates as parameters [40]. Furthermore, a study by Alsabani et al. [41] showed that patients with high SII ratios have an increased risk for longer hospitalization after orthopedic surgeries. The authors also suggested in the same study that those who required hospital stays longer than 21 days are prone to lower hemoglobin levels, increased durations of surgeries and ICU admissions.

This study has a few limitations that need to be taken into consideration. Firstly, it is a retrospective single-center study, which can be improved by changing the nature of the design to a prospective one that includes multiple centers. This may also provide an opportunity to analyze the relationship of these markers to different complications and outcomes after orthopedic surgical interventions. Secondly, a more dynamic approach towards the markers can be adopted by analyzing their behavior through repeated investigations during hospital stays in a standardized manner. Another aspect is that this study did not explain the pathophysiological pathways that stand behind the elevated values of these postoperative inflammatory ratios. Lastly, not all parameters that may have influenced the laboratory data were evaluated, such as diet, hydration state of the patients, chronic medication with anti-inflammatory drugs and blood transfusions or losses.

## 5. Conclusions

This is the first study to investigate the relationship between the magnitude of surgical trauma and inflammatory blood-derived marks in young and middle-aged patients with simple HSFs (AO/OTA 12−A and B). Postoperative NLR > 7.20 and AISI > 1668.58 on the first day are strongly correlated with the invasiveness of the surgical protocol followed. Additionally, operative times higher than 61 min contribute to the post-traumatic stress sustained by the organism.

Considering that these two biologic markers are easy to obtain and cost-effective, with the aid of additional studies, they can be implemented in clinical practice to provide additional indications or contraindications for certain orthopedic programs that are controversial, to identify patients with fractures and even to develop predictive patterns for certain postoperative complications.

## Figures and Tables

**Table 1:** ROC curve analysis presenting the optimal cut-off values of the continuous variables.

Variables	Cut-Off Values	AUC	95% CI	Sensitivity	Specificity	*p* Value
NLR—Admission	4.99	0.398	0.307–0.488	48.5%	35.6%	0.028
PLR—Admission	155.26	0.450	0.358–0.542	47.1%	48.9%	0.281
MLR—Admission	0.63	0.511	0.419–0.603	50.0%	62.2%	0.814
SII—Admission	1428.60	0.415	0.323–0.507	32.4%	54.4%	0.067
SIRI—Admission	142.14	0.503	0.412–0.603	44.1%	58.9%	0.941
AISI—Admission	891.09	0.460	0.368–0.552	48.5%	51.1%	0.393
NLR—Postoperative	7.20	0.780	0.704–0.857	75.0%	75.6%	<0.0001
PLR—Postoperative	174.22	0.700	0.617–0.783	79.4%	61.1%	<0.0001
MLR—Postoperative	0.82	0.707	0.625–0.788	61.8%	68.9%	<0.0001
SII—Postoperative	1564.74	0.797	0.726–0.868	79.4%	72.2%	<0.0001
SIRI—Postoperative	156.95	0.744	0.667–0.821	77.9%	63.3%	<0.0001
AISI—Postoperative	1668.58	0.802	0.731–0.873	70.6%	82.2%	<0.0001
Injury till surgery (days)	1	0.590	0.501–0.680	61.8%	53.3%	0.052
Surgery duration (minutes)	61	0.904	0.858–0.949	82.4%	84.4%	<0.0001
Length of hospitalization (days)	6	0.673	0.589–0.756	80.9%	45.6%	<0.0001

Notes: NLR—neutrophil-per-lymphocyte ratio; PLR—platelet-per-lymphocyte ratio; MLR—monocyte-per-lymphocyte ratio; SII—systemic inflammatory index; SIRI—systemic inflammatory response index; AISI—aggregate inflammatory systemic index; ROC—receiver operating characteristic; AUC—area under the curve; CI—confidence interval. A *p* value < 0.05 was defined as statistically significant.

**Table 2:** Univariate statistical analysis between the two types of surgical protocols performed.

Variable	All Patients (*n* = 158)	Group A (*n* = 90)	Group B (*n* = 68)	*p* Value	Probability Correction Coefficient (r)
**Baseline Characteristics**					
Age (years),	45.39 ± 11.90	44.92 ± 11.82	46.00 ± 11.83	0.571	0.061
mean ± SD					
median (IQR)	49.00 (21)	49.00 (20)	49.00 (22)		
Sex, *n* (%)					
Male	69 (43.7)	33 (47.8)	36 (52.2)	0.051	0.162
Female	89 (56.3)	57 (64.0)	32 (36.0)		
Alcohol (yes), *n* (%)	42 (26.6)	25 (59.5)	17 (40.5)	0.696	−0.031
Smoking (yes), *n* (%)	42 (26.6)	27 (64.3)	15 (35.7)	0.263	−0.089
Overweight (yes), *n* (%)	77 (48.7)	41 (53.2)	36 (46.8)	0.358	0.073
Residence, *n* (%)					
Rural	72 (45.6)	43 (59.7)	29 (40.3)	0.521	0.051
Urban	86 (54.4)	47 (54.7)	39 (45.3)		
Hypertension (yes), *n* (%)	87 (55.1)	51 (58.6)	36 (41.4)	0.641	−0.037
IHD (yes), *n* (%)	47 (29.7)	25 (53.2)	22 (46.8)	0.533	0.050
Asthma (yes), *n* (%)	24 (15.2)	14 (58.3)	10 (41.7)	0.883	−0.012
CB (yes), *n* (%)	22 (13.9)	10 (45.5)	12 (54.5)	0.346	0.093
Type 2 diabetes (yes), *n* (%)	22 (13.9)	17 (56.7)	13 (43.3)	0.971	0.003
**Fracture characteristics**					
AO classification, *n* (%)					
A	77 (48.7)	46 (59.7)	31 (40.3)	0.492	0.055
B	81 (51.3)	44 (54.3)	37 (45.7)		
MoI, *n* (%)					
High	99 (62.7)	56 (56.6)	43 (43.4)	0.896	0.010
Low	59 (37.3)	34 (57.6)	25 (42.4)		
Side of the injury, *n* (%)					
Left	63 (39.9)	38 (60.3)	25 (39.7)	0.488	0.055
Right	95 (60.1)	52 (54.7)	43 (45.3)		
**Surgical factors**					
ASA score, *n* (%)					
I-II	103 (65.2)	60 (58.3)	43 (41.7)	0.654	0.036
≥III	55 (34.8)	30 (54.5)	25 (45.5)		
Type of anesthesia, *n* (%)					
Regional	28 (17.7)	18 (64.3)	10 (35.7)	0.514	0.069
General	130 (82.3)	72 (55.4)	58 (44.6)		
Injury till surgery (days),					
0–1 cut-off	74 (46.8)	48 (64.9)	26 (35.1)	0.060	0.150
>1	84 (53.2)	42 (50.0)	42 (50.0)		
LOH (days),					
0–6 cut-off	54 (34.2)	41 (75.9)	13 (24.1)	0.004	0.276
>6	104 (65.8)	49 (47.1)	55 (52.9)		
Surgery duration (min),					
0–61 cut-off	90 (57.0)	76 (84.4)	14 (15.6)	<0.0001	0.639
>61	68 (43.0)	14 (20.6)	54 (79.4)		
**Laboratory data at admission**					
Neutrophil count (×10^3/^µL), median (IQR)	7.51 (4.49)	7.88 (4.19)	7.30 (4.62)	0.077	0.116
Lymphocyte count (×10^3^/µL), median (IQR)	1.33 (0.75)	1.23 (0.69)	1.48 (0.78)	0.072	0.118
Monocyte count (×10^3^/µL), median (IQR)	0.72 (0.38)	0.70 (0.34)	0.78 (0.43)	0.173	0.090
PLT count (×10^3^/µL), median (IQR)	218 (92)	222.50 (86)	213.50 (98)	0.711	−0.024
AST/ALT (>1, reference), median (IQR)	1.42 (0.59)	1.44 (0.69)	1.39 (0.55)	0.892	−0.009
WBC (×10^3^/µL), median (IQR)	10.20 (4.37)	10.31 (4.48)	9.45 (4.41)	0.353	−0.061
RBC (×10^6^/µL), median (IQR)	3.97 (1.17)	3.87 (1.16)	4.01 (1.18)	0.454	0.055
HGB (g/dL), median (IQR)	12.05 (3.28)	12.05 (3.33)	12.00 (3.06)	0.745	0.012
NLR (>4.99, cut-off), *n* (%)	91 (57.6)	58 (63.7)	33 (36.3)	0.045	0.159
PLR (>155.26, cut-off), *n* (%)	78 (49.4)	46 (59.0)	32 (41.0)	0.614	−0.040
MLR (>0.63, cut-off), *n* (%)	70 (44.3)	36 (51.4)	34 (48.6)	0.210	0.100
SII (>1428.60, cut-off), *n* (%)	63 (39.9)	41 (65.1)	22 (34.9)	0.093	0.136
SIRI (>142.14, cut-off), *n* (%)	67 (42.4)	37 (55.2)	30 (44.8)	0.705	0.030
AISI (>891.09), *n* (%)	77 (48.7)	44 (57.1)	33 (42.9)	0.964	−0.004
**Laboratory data after surgery**					
Neutrophil count (×10^3^/µL), median (IQR)	8.18 (5.76)	6.58 (3.94)	10.16 (4.02)	<0.0001	0.485
Lymphocyte count (×10^3^/µL), median (IQR)	1.11 (0.72)	1.30 (0.79)	1.03 (0.56)	0.007	0.216
Monocyte count (×10^3^/µL), median (IQR)	0.83 (0.50)	0.76 (0.52)	0.91 (0.44)	0.010	0.206
PLT count (×10^3^/µL), median (IQR)	225 (77)	207.00 (86)	243.00 (69)	0.002	0.247
WBC (×10^3^/µL), median (IQR)	9.52 (5.33)	8.87 (3.71)	11.27 (5.57)	<0.0001	0.295
RBC (×10^6/^µL), median (IQR)	3.04 (0.94)	3.07 (0.92)	2.96 (0.96)	0.664	−0.048
HGB (g/dL), median (IQR)	9.20 (2.07)	9.25 (2.11)	8.95 (2.27)	0.467	−0.028
NLR (>7.20, cut-off), *n* (%)	73 (46.2)	22 (30.1)	51 (69.9)	<0.0001	0.508
PLR (>174.22, cut-off), *n* (%)	89 (56.3)	35 (39.3)	54 (60.7)	<0.0001	0.405
MLR (>0.82, cut-off), *n* (%)	72 (45.6)	30 (41.7)	42 (58.3)	<0.0001	0.283
SII (>1564.74, cut-off), *n* (%)	77 (48.7)	24 (31.2)	53 (68.8)	<0.0001	0.502
SIRI (>156.96, cut-off), *n* (%)	86 (54.4)	33 (38.4)	53 (61.6)	<0.0001	0.410
AISI (> 668.58), *n* (%)	64 (40.5)	16 (25.0)	48 (75.0)	<0.0001	0.533

Notes: IHD—ischemic heart disease; CB—chronic bronchitis; AO—Arbeitsgemeinschaft für Osteosynthesefragen; MoI—mechanism of injury; ASA score—American Society of Anesthesiologists score; LOH—length of hospitalization; PLT—platelet counts; AST—aspartate−transaminase; ALT—alanine−transaminase; WBC—white blood counts; RBC—red blood counts; HGB—hemoglobin; NLR—neutrophil-per-lymphocyte ratio; PLR—platelet-per-lymphocyte ratio; MLR—monocyte-per-lymphocyte ratio; SII—systemic inflammatory index; SIRI—systemic inflammatory response index; AISI—aggregate inflammatory systemic index. A *p* value < 0.05 was defined as statistically significant.

**Table 3:** Multivariate statistical analysis of surgical trauma magnitude.

Variable	Magnitude of Surgical Trauma		*p* Value
	OR	95% CI	
NLR Postoperative	17.12	2.14–136.83	**0.007**
PLR Postoperative	1.03	0.24–4.36	0.961
MLR Postoperative	0.67	0.15–2.84	0.590
SII Postoperative	0.26	0.24–3.02	0.287
SIRI Postoperative	1.64	0.22–12.13	0.627
AISI Postoperative	16.15	2.08–125.44	0.008
Days till surgery	1.08	0.29–3.96	0.900
Duration of surgery (min)	76.42	14.41–405.62	<0.0001
LOHS (days)	2.57	0.66–9.93	0.170

Notes: NLR—neutrophil-per-lymphocyte ratio; PLR—platelet-per-lymphocyte ratio; MLR—monocyte-per-lymphocyte ratio; SII—systemic inflammatory index; SIRI—systemic inflammatory response index; AISI—aggregate inflammatory systemic index; LOH—length of hospitalization. A *p* value < 0.05 was defined as statistically significant.

## Data Availability

The data used in this study can be requested from the corresponding author.
